# Supplementary material for: Environmental conditions regulate the impact of plants on cloud formation
Source: Nat Commun. 2017 Feb 20;8:14067. doi: 10.1038/ncomms14067 (PMC5321720; doi:10.1038/ncomms14067)
Supplement: Supplementary Information — Supplementary Figures, Supplementary Tables and Supplementary References [file ncomms14067-s1.pdf]

Supplementary Figures

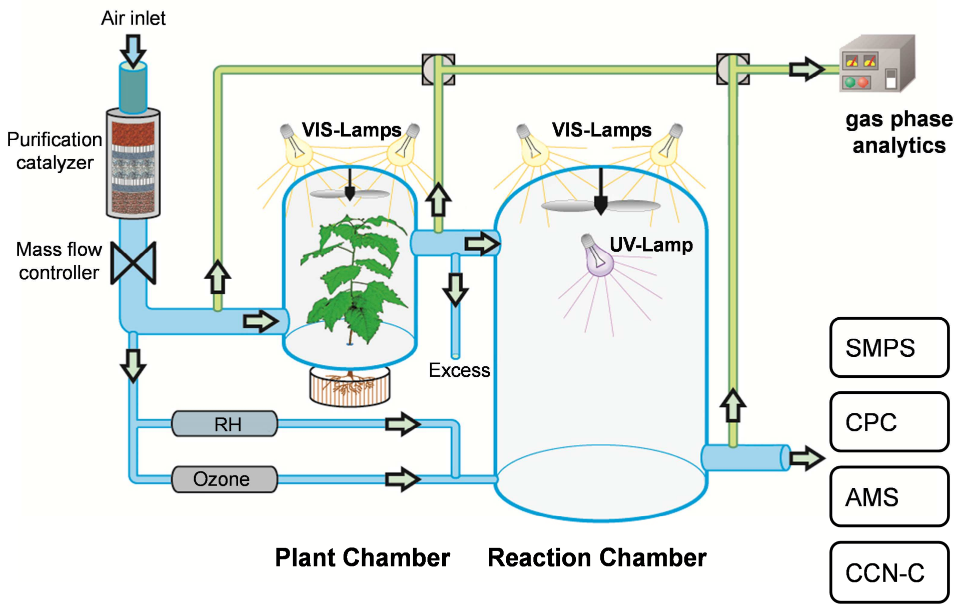

Supplementary Figure 1. **Schematic of the experimental setup.** Juelich Plant Atmosphere Chamber (JPAC) is shown. SMPS: scanning mobility particle sizer; CPC: condensation particle counter; AMS: aerosol mass spectrometer; CCN-C: cloud condensation nuclei counter; RH: relative humidity; Vis-lamp: visible light lamps.

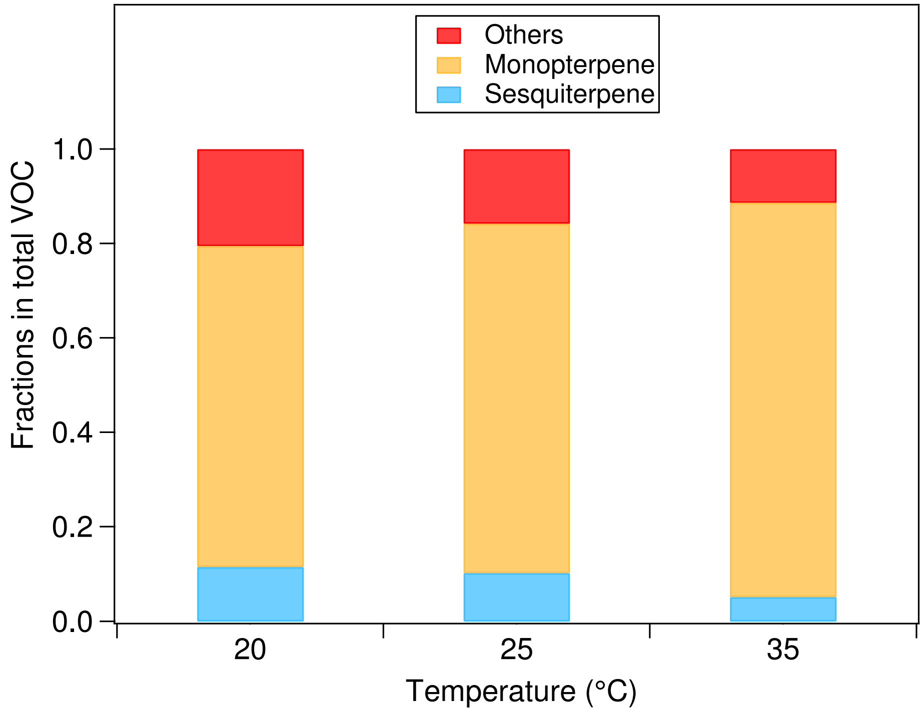

Supplementary Figure 2. **Volatile organic compounds composition of unstressed trees.** Fractions of different classes of volatile organic compounds (VOC) in the total VOC emissions (ppbC) from pines in the absence of biotic stresses at different plant temperatures are shown. Blue, orange and red indicate the fractions of sesquiterpene, monotperene and others (see Methods for detailed classification).

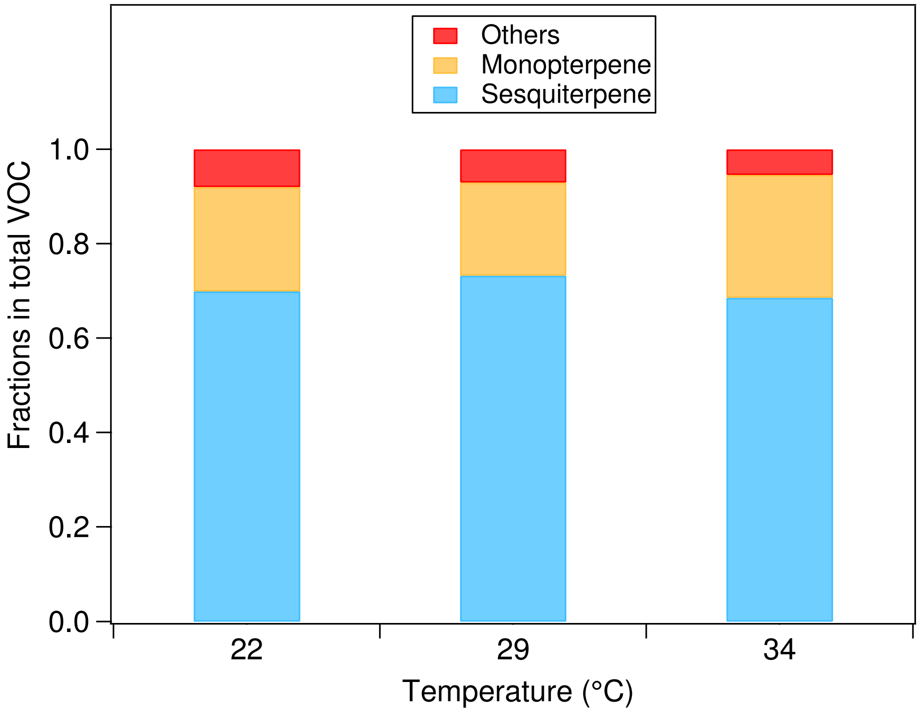

Supplementary Figure 3. **Volatile organic compounds composition of biotically-stressed trees**. Fractions of different classes of volatile organic compounds in total VOC emissions from boreal forest trees under biotic stresses at different plant temperature are shown. Blue, orange and red indicate the fractions of sesquiterpene, monotperene and others (see Methods for detailed classification).

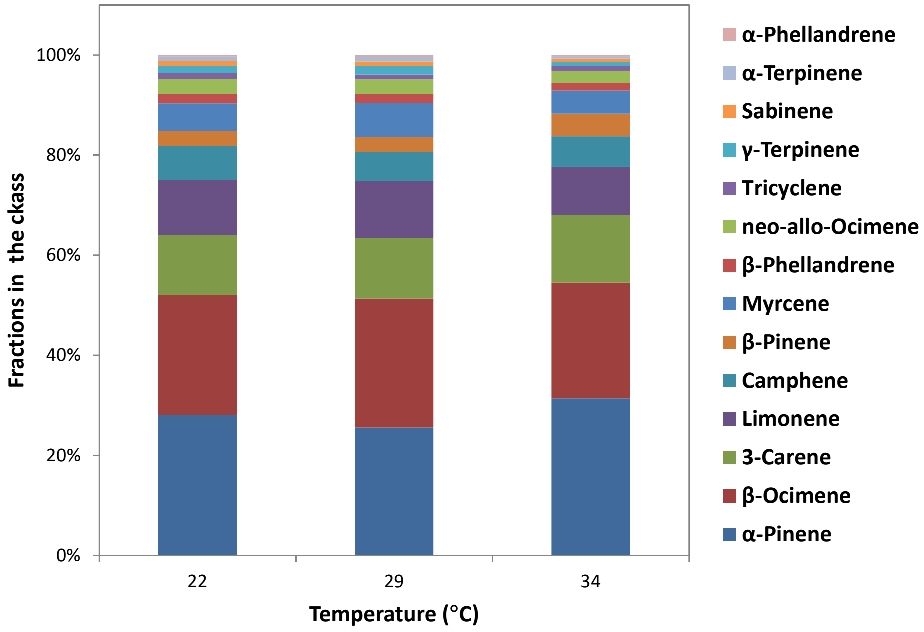
Fractions in the ckass
100%
80%
60%
40%
20%
0%
22
29
34
Temperature (°C)
α-Phellandrene
α-Terpinene
Sabinene
γ-Terpinene
Tricyclene
neo-allo-Ocimene
β-Phellandrene
Myrcene
β-Pinene
Camphene
Limonene
3-Carene
β-Ocimene
α-Pinene

(a)

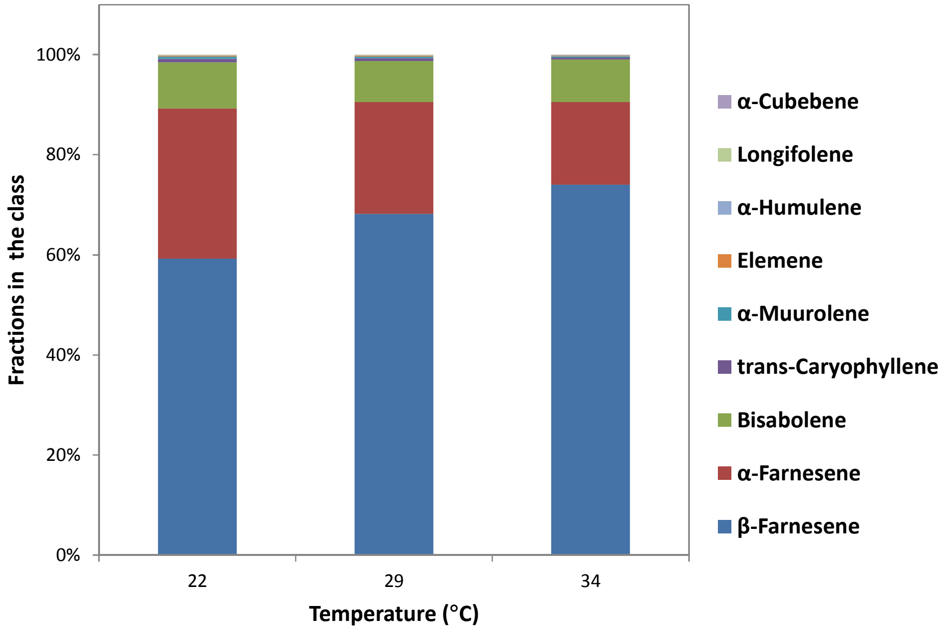
Fractions in the class
100%
80%
60%
40%
20%
0%
22
29
34
Temperature (°C)
α-Cubebene
Longifolene
α-Humulene
Elemene
α-Muurolene
trans-Caryophyllene
Bisabolene
α-Farnesene
β-Farnesene

(b)

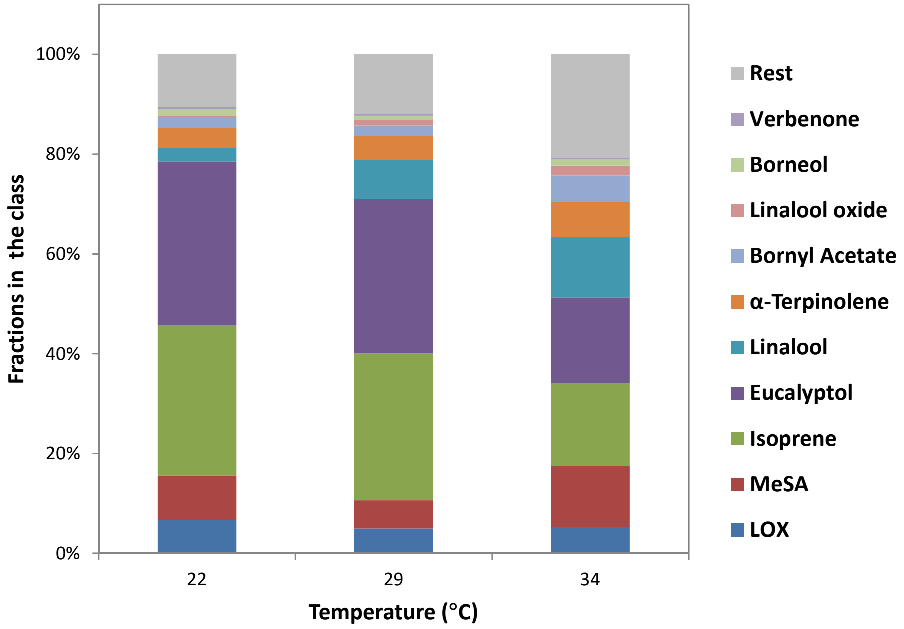

(c)

Supplementary Figure 4. **Detailed volatile organic compounds composition at different temperature.** Fractions of different compounds in each classes for monoterpenes (a), sesquiterpenes (b), and other compounds (c) (see Supplementary Figure 3) for boreal forest trees under biotic stresses at different plant temperature are shown. See the Methods for the details of volatile organic compounds classification.

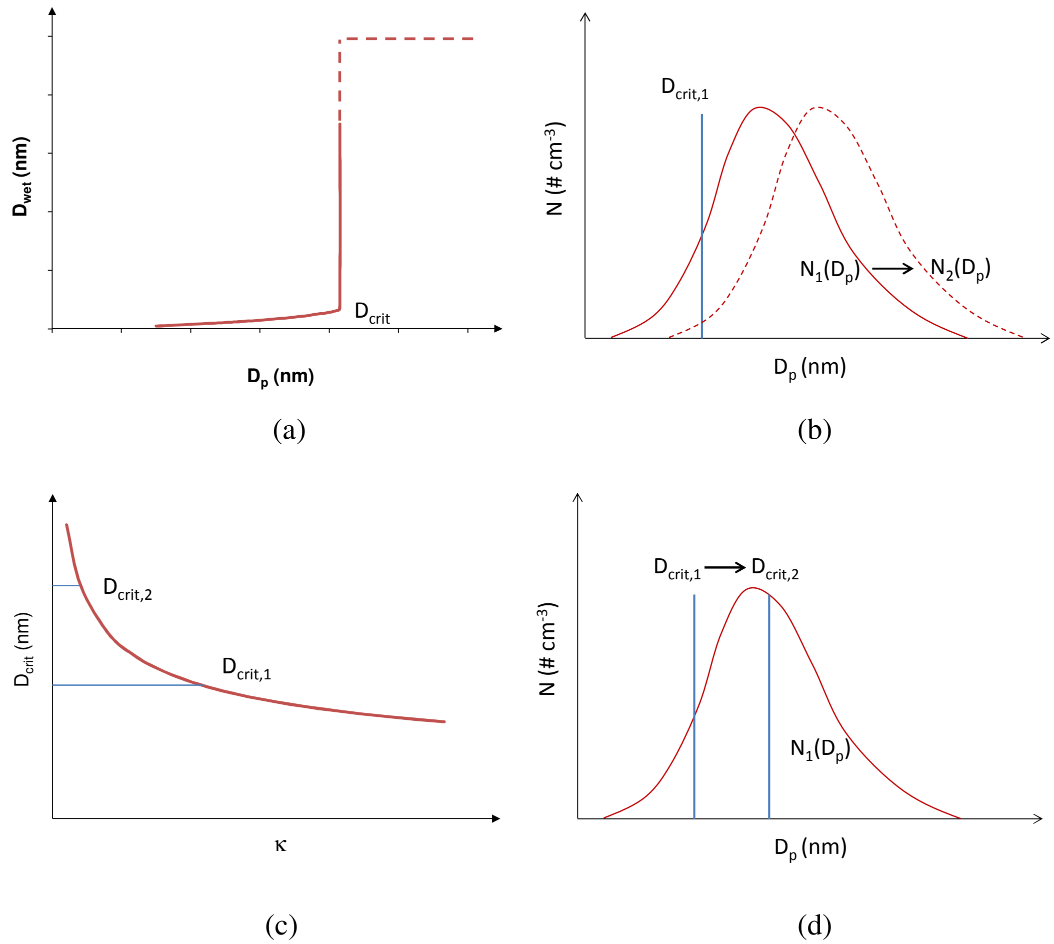

Supplementary Figure 5. **Effects of particle size and κ on cloud condensation nuclei concentrations**. Panel a shows the size of the cloud droplet (D_{wet}) that at given supersaturation (SS=RH-100%) a dry particle of given chemical composition can grow to from subsaturated condition as a function of particle size. The dash line indicates that the final droplet size is uncertain, depending on dynamic conditions in clouds. D_{crit} is the critical activation diameter, which is defined as the particle size above which the droplet formed can grow spontaneously to large droplets and the particle is considered to have activated into a cloud droplet. Panel b shows that for given particle numbers as a function of size (particle size distribution, solid line), how cloud condensation nuclei (CCN) concentration is derived as the total number of particles larger than the critical activation diameter $D_{crit,1}$, i.e. the integral of the part of the size distribution $N_1(D_p)$ that is larger than $D_{crit,1}$ (blue line). The dash line shows a larger particle size distribution $N_2(D_p)$ that the particle size distribution shifts from $N_1(D_p)$. Panel c shows D_{crit} as a function of κ at a given supersaturation. The blue lines indicates the critical diameter $D_{crit,1}$ and $D_{crit,2}$ for a higher and lower κ, respectively.

Panel d shows the effect of an increase of D_{crit} from $D_{crit,1}$ to $D_{crit,2}$ on the CCN concentration (number of particles larger than D_{crit}). The blue lines indicate $D_{crit,1}$ and $D_{crit,2}$, respectively.

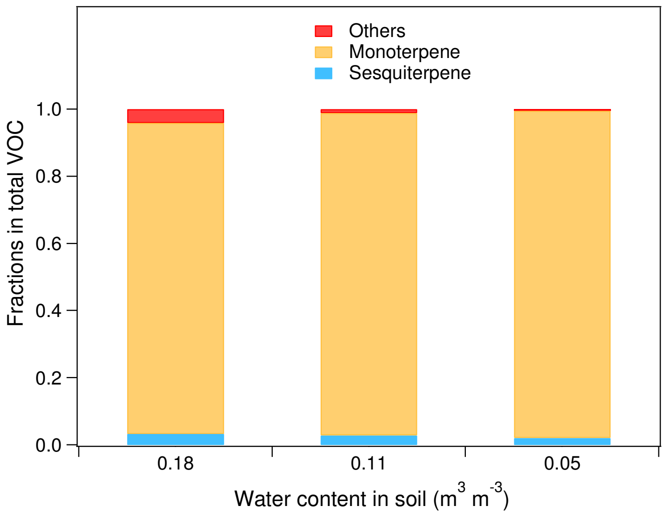

(a)

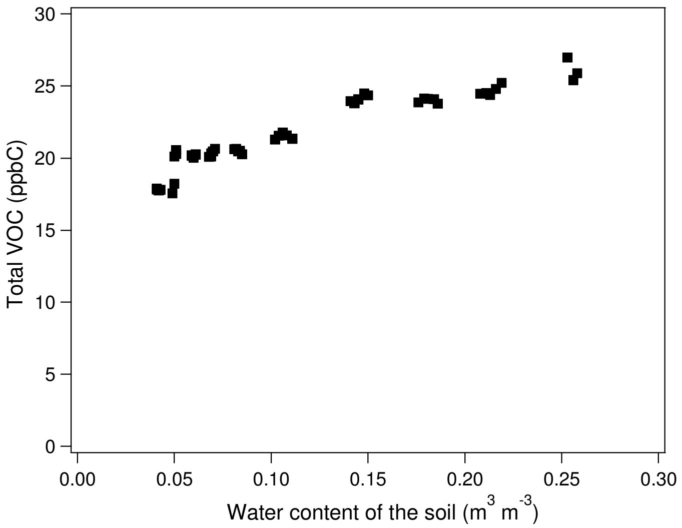

(b)

Supplementary Figure 6. **Effect of drought on volatile organic compounds emissions**. (a). Fractions of different classes of volatile organic compounds (VOC) in total VOC emissions from a pine at different levels of water shortage. The blue, orange and red indicate the fractions of sesquiterpene, monotperene and others (see Methods for detailed classification). (b). The concentration of total VOC (ppbC) emitted as a function of water content in the soil.

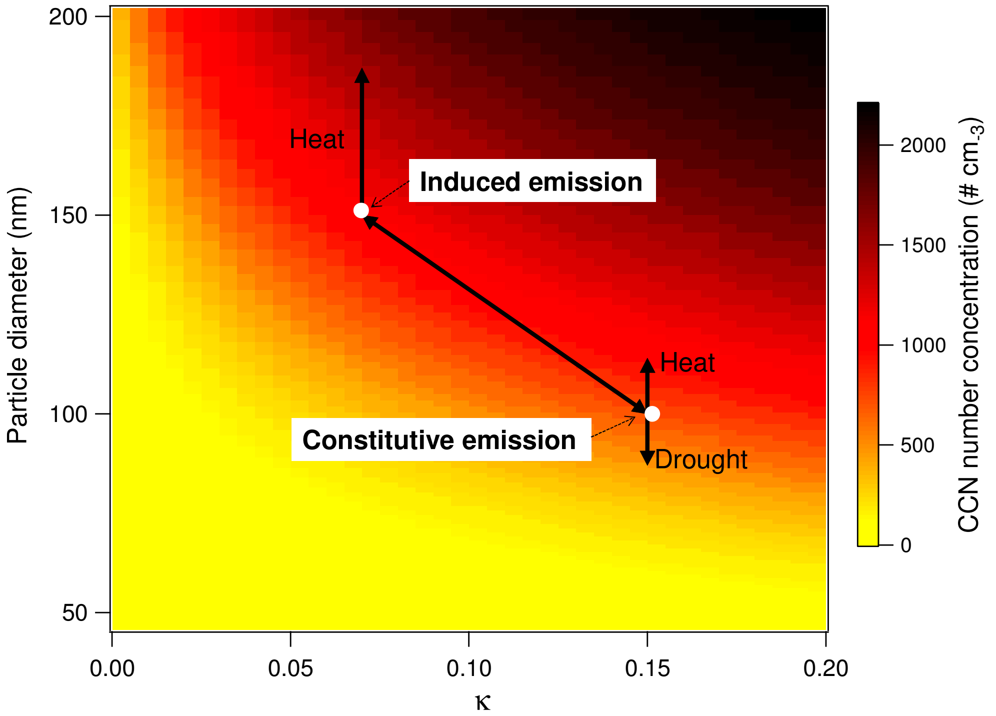

Supplementary Figure 7. **Impact of various stresses on cloud condensation nuclei concentrations.** The effects of biotic stress, heat and drought on κ and particle size and cloud condensation nuclei (CCN) concentrations are shown on a typical plot of CCN number concentrations as a function of size and κ. The effects were calculated based on the results in this study (see Methods for details). The left higher and right lower white dots indicate the reference case of induced emissions and constitutive emissions at room temperature, respectively.

Supplementary Tables

Supplementary Table 1. Summary of κ of SOA from sesquiterpene oxidation from CCN measurements in the literature.

Literature	Precursor	κ value	Oxidation condition	Chamber/Conditions	Comments
Hartz et al.[1]	β-caryophyllene, α-humulene, α-cedrene	0.001-0.009[a]	Ozonolysis	CMU 10 m^3 chamber. VOC: 50-75 ppb, O_3:100-600 ppb, RH: 5-8%	κ was not reported in the paper[e].
Asa-Awuku et al.[2]	β-caryophyllene	0.02-0.05[a]<br>0.01-0.05[b]	Ozonolysis	CMU 12 m^3 chamber. VOC: 22-32 ppb, O_3: 300 ppb, RH: 3-8%, T: 22 °C. NO_x not reported.	κ was not reported in the paper.
Alfarra et al.[3]	β-caryophyllene	0-0.02	Photooxidation	Manchester Univ. 18 m^3 chamber. NO_x: 26.8-125 ppb, VOC: 31.1-139.9 ppb. RH: ~70%	-
Tang et al.[4]	β-caryophyllene	0.05-0.073[a]<br>0.16-0.22[b]	Ozonolysis	UCI 90 m^3 chamber. VOC: 5-20 ppb, O_3: 100-290 ppb, NO_x: not reported. RH: 0.1%	Light has negligible effects on κ.
Frosch et al.[5]	β-caryophyllene	0.001-0.005[a]<br>0.003-0.05[b]<br>0.01-0.075[c]<br>0.04-0.09[d]	Ozonolysis and Photooxidation	PSI 27 m^3 chamber. VOC: 25 ppb O_3: 300 ppb, RH: 5-10%	κ was found to depend on SS.
Alfarra et al.[6]	β-caryophyllene	0.009	Photooxidation	Manchester Univ. 18 m^3 chamber. NO_x: ~25 ppb, VOC: 50/250 ppb. RH: 44-71%	-

[a]: The value is for SOA from ozonolysis with OH scavenger (usually 2-butanol was used).
[b]: The value is for SOA from ozonolysis without OH scavenger .
[c]: The value is for SOA from photooxidation (OH from O_3 photolysis and O_3 reaction).
[d]: The value is for SOA from photooxidation (OH from O_3 photolysis and O_3 reaction plus OH from HONO photolysis).
[e]: κ was calculated using supersaturation and critical activation diameter and a temperature of 298.15 K when κ was not reported in original papers.

Supplementary Table 2. Summary of κ of SOA from monoterpene oxidation from CCN measurements in the literature.

Literature	Precursor	κ value	Oxidation condition	Reactor	Comments
VanReken et al.[7]	α-pinene	0.014-0.091[a]	Ozonolysis	CIT 28 m^3 chamber	κ was not reported in the paper[i].
Hartz et al.[1]	α-pinene	0.028-0.229[a]	Ozonolysis	CMU 10^3 chamber	κ was not reported in the paper.
Prenni et al.[8]	α-pinene	0.1	Ozonolysis	UCR $7m^3$ chamber	-
Engelhart et al.[9]	α-pinene	0.11-0.14[b]	Ozonolysis	CMU 12 m^3 chamber	κ was not reported in the paper.
Duplissy et al.[10]	α-pinene	0.09-0.12	Photooxidation	PSI $27m^3$ chamber	-
Wex et al.[11]	α-pinene	0.1±0.04	Ozonolysis	12L flow reactor	κ was not reported in the paper.
Juranyi et al.[12]	α-pinene	0.091±0.01	Photooxidation	PSI $27m^3$ chamber	-
Massoli et al.[13]	α-pinene	0.13-0.24[c]	Photooxidation	Aerosol flow reactor	-
Frosch et al.[14]	α-pinene	0.06-0.16[c]	Photooxidation	PSI $27m^3$ chamber	-
Lambe et al.[15]	α-pinene	0.12-0.23[c]	Photooxidation	15L PAM flow reactor	-
Lambe et al.[16]	α-pinene	0.12-0.18	Photooxidation	15L PAM flow reactor	-
Alfarra et al.[6]	α-pinene	0.10-0.17	Photooxidation	Univ. Manchester $18m^3$ chamber	-
Zhao et al.[17]	α-pinene	0.10-0.17	Photooxidation	FZJ 270 m^3 chamber	
Hartz et al.[1]	β-pinene	0.033-0.106[d]<br>0.044[e]	Ozonolysis	CMU 10^3 chamber	κ was not reported in the paper.
VanReken et al.[7]	β-pinene	0.02-0.18[e, f]	Ozonolysis	CIT 28 m^3 chamber	κ was not reported in the paper.
Prenni et al.[8]	β-pinene	0.07-0.10[e]	Ozonolysis	UCR $7m^3$ chamber	κ was not reported in the paper.
Hartz et al.[1]	limonene	0.017-0.44[d]<br>0.170[e]	Ozonolysis	CMU 10^3 chamber	κ was not reported in the paper.
VanReken et al.[7]	limonene	0.05-0.45[e, f]	Ozonolysis	CIT 28 m^3 chamber	κ was not reported in the paper.
Alfarra et al.[6]	limonene	0.06[g]	Photooxidation	Univ. Manchester $18m^3$ chamber	-
Hartz et al.[1]	carene	0.02-0.13[e]	Ozonolysis	CMU 10^3 chamber	κ was not reported in the paper.
VanReken et al.[7]	carene	0.04-0.25[e, f]	Ozonolysis	CIT 28 m^3 chamber	κ was not reported in the paper.
Prenni et al.[8]	carene	0.07-0.13[e]	Ozonolysis	UCR $7m^3$ chamber	κ was not reported in the paper.
Alfarra et al.[6]	myrcene	0.10-0.12	Photooxidation	Univ. Manchester $18m^3$ chamber	-
Engelhart et al.[9]	monoterpene mixtures[h]	0.08-0.27	Ozonolysis	CMU 12 m^3 chamber	κ was not reported in the paper.
Zhao et al.[18]	α-pinene/limonene mixture	0.10-0.17	Photooxidation	FZJ 270 m^3 chamber	

[a]: κ here was from Petters and Kreidenweis[19].

[b]:κ here was from Engelhart et al.[20]

[c]: Values were not explicitly provided and were read from the figures in the paper.

[d]: The value is for SOA from ozonolysis with OH scavenger (usually 2-butanol was used).

[e]: The value is for SOA from ozonolysis without OH scavenger .

[f]: D_{crit} and SS data are read from the figures in the paper.

[g]: The authors note that the value is derived from a limited number of measurements.

[h]: Monoterpene mixtures consist of α-pinene, β-pinene, limonene and carene.

[i]: κ was calculated using supersaturation and critical activation diameter data and a temperature of 298.15 K when κ was not reported in original papers except otherwise stated.

Supplementary Table 3. Number of measurements and measured ratios of monoterpene to sesquiterpene and κ in Figure. 2.

Stress status	N(MT/SQT)	N(κ)	MT/SQT	κ
Unstressed	4	3	8.37±1.55[a]	0.15±0.02
Intermediate	4	6	2.74±0.35	0.09±0.01
Stressed	20	48	0.32±0.05	0.07±0.01

[a]: Standard deviation.

MT: monoterpene; SQT: sesquiterpene; N(MT/SQT) and N(κ): number of measurements for the ratios of monoterpene to sesquiterpene and for κ, respectively.

Supplementary Table 4. Number of measurements and measured total VOC, particle size and κ in Figure. 3.

Emission type	Temperature (°C)	N(VOC)	N(size)	N(κ)	TVOC (ppbC)	Size (nm)	κ
Constitutive emission	20	6	6	-	19.2±2.1[a]	28.9±0.7	-
	25	4	6	3	40.3±6.2	55.0±2.3	0.15±0.02
	35	2	3	4	84.2±9.6	69.8±1.0	0.11±0.03
Induced emission	22	19	15	48	83.5±19.3	60.2±8.3	0.073±0.01
	29	12	9	11	236.4±24.2	85.7±1.5	0.065±0.01
	34	8	6	15	731.4±243.0	116.1±5.6	0.059±0.01

[a]: Standard deviation.

N(VOC), N(size), N(κ): number of measurements for total VOC, particle size and κ, respectively.

Supplementary References

1. Hartz, K. E. H. *et al.* Cloud condensation nuclei activation of monoterpene and sesquiterpene secondary organic aerosol. *J. Geophys. Res.-Atmos.* **110**, D14208 (2005).

2. Asa-Awuku, A., Engelhart, G. J., Lee, B. H., Pandis, S. N. & Nenes, A. Relating CCN activity, volatility, and droplet growth kinetics of beta-caryophyllene secondary organic aerosol. *Atmos. Chem. Phys.* **9**, 795-812 (2009).

3. Alfarra, M. R. *et al.* The effect of photochemical ageing and initial precursor concentration on the composition and hygroscopic properties of beta-caryophyllene secondary organic aerosol. *Atmos. Chem. Phys.* **12**, 6417-6436 (2012).

4. Tang, X., Cocker, D. R., III & Asa-Awuku, A. Are sesquiterpenes a good source of secondary organic cloud condensation nuclei (CCN)? Revisiting beta-caryophyllene CCN. *Atmos. Chem. Phys.* **12**, 8377-8388 (2012).

5. Frosch, M. *et al.* CCN activity and volatility of beta-caryophyllene secondary organic aerosol. *Atmos. Chem. Phys.* **13**, 2283-2297 (2013).

6. Alfarra, M. R. *et al.* Water uptake is independent of the inferred composition of secondary aerosols derived from multiple biogenic VOCs. *Atmos. Chem. Phys.* **13**, 11769-11789 (2013).

7. VanReken, T. M., Ng, N. L., Flagan, R. C. & Seinfeld, J. H. Cloud condensation nucleus activation properties of biogenic secondary organic aerosol. *J. Geophys. Res.-Atmos.* **110**, D07206 (2005).

8. Prenni, A. J., Petters, M. D., Kreidenweis, S. M., DeMott, P. J. & Ziemann, P. J. Cloud droplet activation of secondary organic aerosol. *J. Geophys. Res.-Atmos.* **112**, D10223 (2007).

9. Engelhart, G. J., Asa-Awuku, A., Nenes, A. & Pandis, S. N. CCN activity and droplet growth kinetics of fresh and aged monoterpene secondary organic aerosol. *Atmos. Chem. Phys.* **8**, 3937-3949 (2008).

10. Duplissy, J. *et al.* Cloud forming potential of secondary organic aerosol under near atmospheric conditions. *Geophys. Res. Lett.* **35**, L03818 (2008).

11. Wex, H. *et al.* Towards closing the gap between hygroscopic growth and activation for secondary organic aerosol: Part 1-Evidence from measurements. *Atmos. Chem. Phys.* **9**, 3987-3997 (2009).

12. Juranyi, Z. *et al.* Influence of gas-to-particle partitioning on the hygroscopic and droplet activation behaviour of alpha-pinene secondary organic aerosol. *Phys. Chem. Chem. Phys.* **11**, 8091-8097 (2009).

13. Massoli, P. *et al.* Relationship between aerosol oxidation level and hygroscopic properties of laboratory generated secondary organic aerosol (SOA) particles. *Geophys. Res. Lett.* **37**, L24801 (2010).

14. Frosch, M. *et al.* Relating cloud condensation nuclei activity and oxidation level of alpha-pinene secondary organic aerosols. *J. Geophys. Res.-Atmos.* **116**, D22212 (2011).

15 Lambe, A. T. *et al.* Laboratory studies of the chemical composition and cloud condensation nuclei (CCN) activity of secondary organic aerosol (SOA) and oxidized primary organic aerosol (OPOA). *Atmos. Chem. Phys.* **11**, 8913-8928 (2011).

16 Lambe, A. T. *et al.* Characterization of aerosol photooxidation flow reactors: heterogeneous oxidation, secondary organic aerosol formation and cloud condensation nuclei activity measurements. *Atmos. Meas. Tech.* **4**, 445-461 (2011).

17 Zhao, D. F. *et al.* Size-dependent hygroscopicity parameter (κ) and chemical composition of secondary organic cloud condensation nuclei. *Geophys. Res. Lett.* **42**, 10920-10928 (2015).

18 Zhao, D. F. *et al.* Cloud condensation nuclei activity, droplet growth kinetics, and hygroscopicity of biogenic and anthropogenic secondary organic aerosol (SOA). *Atmos. Chem. Phys.* **16**, 1105-1121 (2016).

19 Petters, M. D. & Kreidenweis, S. M. A single parameter representation of hygroscopic growth and cloud condensation nucleus activity. *Atmos. Chem. Phys.* **7**, 1961-1971 (2007).

20 Engelhart, G. J., Moore, R. H., Nenes, A. & Pandis, S. N. Cloud condensation nuclei activity of isoprene secondary organic aerosol. *J. Geophys. Res.-Atmos.* **116**, D02207 (2011).
